# Sex knowledge, attitudes, and high-risk sexual behaviors among unmarried youth in Hong Kong

**DOI:** 10.1186/1471-2458-13-691

**Published:** 2013-07-29

**Authors:** Paul SF Yip, Huiping Zhang, Tai-Hing Lam, Kwok Fai Lam, Antoinette Marie Lee, John Chan, Susan Fan

**Affiliations:** 1 Department of Social Work and Social Administration, The University of Hong Kong, Hong Kong, Hong Kong; 2 Center for Suicide Research and Prevention, The University of Hong Kong, Hong Kong, Hong Kong; 3 Department of Social Work, The School of Sociology and Population Studies, Renmin University of China, Beijing, P.R. China; 4 Department of Community Medicine, School of Public Health, The University of Hong Kong, Hong Kong, Hong Kong; 5 Department of Statistics & Actuarial Science, The University of Hong Kong, Hong Kong, Hong Kong; 6 Department of Psychiatry, Li Ka Shing Faculty of Medicine, The University of Hong Kong, Hong Kong, Hong Kong; 7 Family Planning Association of Hong Kong, Wan Chai, Hong Kong

## Abstract

**Background:**

Little is known about sex knowledge, attitudes, and high-risk sexual behaviors among unmarried youth in Hong Kong. It is of public health importance to investigate this topic to inform sex education, policymaking, and prevention and intervention programs.

**Methods:**

Based on the Youth Sexuality Survey conducted by Hong Kong Family Planning Association (FPAHK) in 2011, this study explored the characteristics of sexual knowledge, attitudes, and high-risk sexual behaviors among 1,126 unmarried youth aged 18 to 27 years. Multiple logistic regressions were performed to examine factors associated with unmarried youth’s premarital sex, casual relationships, multiple sex partners, and premarital pregnancy.

**Results:**

Unmarried youth in Hong Kong had adequate sex knowledge, but contraceptive knowledge was deficient. The majority of unmarried youth (63.8%) held liberal attitudes toward premarital sex and about half held liberal attitudes toward any form of sexual activity and premarital pregnancy. Around 60% held conservative attitudes toward causal sex relationships and multiple sex partners. Males tended to hold more liberal attitudes toward high-risk sex behaviors than female youth. Approximately 41.5% of unmarried youth reported having engaged in premarital sex, whereas less than 10% engaged in high-risk sexual behaviors. Males also reported higher amounts of premarital sex, casual sex relationships, and multiple sex partners. Females reported higher levels of sexual coercion. Logistic regressions indicated that being older, coming from a divorced family, out of school status and liberal attitudes toward risky sex behavior were more likely to engage in premarital sex or high-risk sex behaviors, and being female, being better educated and being immigrants were less likely to engage in premarital sex. However, being immigrants was more likely to engage in casual relationship and to have multiple partners.

**Conclusions:**

Premarital sex is becoming more prevalent among unmarried youth in Hong Kong, and a small proportion of young adults are engaging in high-risk sexual behaviors. Sex education and HIV prevention programs should equip them with adequate knowledge on contraception and condom use. Intervention programs can start with their attitudes toward sex.

## Background

Hong Kong, as an international and the most westernized city in China, has been significantly influenced by both Western and Chinese cultures. Due to some 150 years of British governance, Hong Kong has integrated many Western ideas and practices, but still maintained their Chinese identity [1]. Attitudes toward sex have been changing dramatically, with premarital sex and even high-risk sexual behaviors not uncommon among young people in Hong Kong. For example, according to the Youth Sexuality Survey of the Family Planning Association of Hong Kong, the prevalence of premarital sex has increased from 35.1% in 1996 to 44% in 2006 among unmarried males, from 27.5% in 1996 to 31.0% among unmarried females aged 18 to 27 years [2]. High-risk sexual behaviors (e.g., multiple partners, having casual or unknown partners, and failing to take protective actions during sexual intercourse) among unmarried young adults in Hong Kong have also been reported [3,4]. Premarital sex and high-risk sexual behaviors not only increases the possibility of negative consequences that may endanger health (e.g., HIV/AIDS or other sexually transmitted infections [STIs]), but also result in higher rates of unplanned pregnancy [5,6].

Among researchers and prevention and intervention programs, young people have always been considered most vulnerable to HIV infections. The United Nations estimated that nearly one of every two new HIV infections worldwide occurs within young adults between 15 and 24 years old [7]. In the US, about half of the sexually active youth acquires a STI by the age of 25 years [8]. Similarly, over 60% of all HIV infections in China occur within young people between 15 and 29 years old [9]. Therefore, it is of great public health importance to investigate sex knowledge, attitudes, and behaviors among unmarried youth. Doing so provides evidence for effective delivery of youth health services and guides public health policymaking.

Most studies investigating young people’s sexual knowledge, attitudes, and behaviors in Hong Kong have been conducted on adolescents and college students. In general, adolescents aged 15 to 17 years showed a low level of sex knowledge, especially with regard to birth control, STIs, and the probability of pregnancy [10]. According to a 1995 survey on 15- to 29-year-olds in Hong Kong, 40% of sexually active youths did not use condoms and 20% of young men had visited sex workers [11]. Another study showed that over 60% of Hong Kong students studying in US colleges approved of premarital sexual intercourse when the partners were in love or engaged [12]. However, compared with their counterparts in the US, Hong Kong Chinese college students were sexually less experienced and more conservative [13]. It is important to note that all these studies were conducted more than ten years ago and most of the samples were not population-based, thus they not only fail to capture a comprehensive picture, but also cannot provide updated evidence that could be used for the prevention of, and intervention on, unmarried youth’s sexual behavior.

To effectively prevent HIV/AIDS infections and other health-endangering consequences, many studies have aimed to identify risk factors of premarital sex and high-risk sex behaviors among unmarried youth. Age is associated with sexual intercourse: older adults had significantly higher probability of sexual intercourse [14]. Gender is also a predictor of premarital sex and high-risk sex behavior, with males more likely to participate in sexual intercourse than females [15,16]. Out of school status is associated with increased sexual activity, unprotected sex, and pregnancy [17,18]. Immigration status also plays a role in young adults’ sex behavior: Immigrant youth are more likely to engage in sexual behavior and risky behavior [19]. Family context also strongly impacts young people’s sexual behavior, with an intact family preventing youth from participating in premarital sex [20]. In addition, previous studies have demonstrated that youth’s attitudes toward sex influence their sexual behavior, with more liberal attitudes leading to premarital sex and high-risk sex behaviors [21,22]. Surprisingly, few studies have explored the influences of these factors on premarital sex and high-risk sex behaviors among unmarried youth in Hong Kong.

Compared to the rich knowledge base in Western countries, little is known about the characteristics of sexual knowledge, attitudes, and behavior among unmarried youth (and the associated factors for high-risk sex behaviors) in Hong Kong. Based on the Youth Sexuality Study of the Hong Kong Family Planning Association in 2011, the purpose of this representative study aimed to address three research questions: (a) What are unmarried youth’s sex knowledge and attitudes toward premarital sex and high-risk sexual behavior?; (b) What is the prevalence of premarital sex and high-risk sexual behavior?; and (c) What are the associated factors of premarital sex and risky sex behaviors among unmarried youth in Hong Kong? Such information is necessary for health professionals to prevent and intervene on not only unmarried young adults’ sexual behavior, but also other negative consequences.

## Methods

### Participants and procedure

This study only used the unmarried youth data from the Youth Sexuality Study (YSS) conducted by the Family Planning Association of Hong Kong (FPAHK) from September to December in 2011. The YSS is the longest-running, community-based household survey of youth sexuality in Hong Kong, which has been carried out every 5 years since 1981. It assesses youth’s sex knowledge, attitudes, experiences, and family relationships.

The Hong Kong Census and Statistic Department (HKCSD) provided a systemically selected, random sample of 11,518 living quarters. A total of 9,654 households were successfully contacted, with 13.7% having youths aged 18 to 27 years. If the household had youths in the required age group, the fieldworker would ask whether the youths were available or not. If the household had youths but were not present then they re-visited. If only one youth was present, then they asked the youth to participate the interview even the household had more than one youth. If more than one youth were present, then they used the nearest birthday method to pick one. Finally, 1,223 participants completed the survey, yielding a response rate of 80%. After excluding those married, 1,126 unmarried youth (594 males and 532 females) aged 18 to 27 years old were included in this study.

Participants were identified and invited to participate in the survey by the experienced fieldworkers. A pilot survey (*N* = 40) was conducted in September 2011 to test the flow of the questionnaire. The questionnaire was then revised before fieldwork adoption. If there were eligible respondents, face-to-face interviews were conducted unless the respondents requested to do the questionnaires by themselves. The questionnaires were anonymous and confidentiality was emphasized. The fieldworkers recorded contact results to assess response rate. If the respondents could not be contacted, a maximum of 5 revisits were attempted before being classified as unsuccessful. The fieldwork was completed in December 2011.

The standardized questionnaire collected information on the participant’s age, gender, education, immigration status ( *yes* or *no*), current school status (*in* or *out of school*), parents’ marital status (*married*, *divorced*, or *deceased*), family life satisfaction (*very satisfied*, *satisfied*, *less than satisfied*), attitudes toward different kinds of high-risk sexual behaviors (e.g., premarital sex, any kinds of mutually agreed upon sexual activities, premarital pregnancy, compensated dating with sex, and multiple sex partners). Sex-related knowledge and high-risk sexual behaviors were also assessed.

### Measures

This study included 12 items pertaining to sex-related knowledge (e.g., contraception, sexually transmitted disease (STD), and HIV/STD transmission). Three composite scores were created by adding the number of correct answers to the four questions on contraception (1 = the chance of pregnancy is slim two weeks before menses; 2 = the chance of pregnancy is slim in first intercourse; 3 = no pregnancy if there is no orgasm; 4 = pregnancy can occur with extravaginal ejaculation), four questions on STD (1 = condom reduces the chance of getting venereal disease; 2 = multiple sex partners increases chances of venereal disease; 3 = venereal disease affects only sexual organs; 4 = venereal disease can be unknown to its carriers), and four questions on HIV/STD transmission (1 = only homosexuals can get AIDS, 2 = AIDS can be unknown to its carriers; 3 = multiple sexual partners increases chances of AIDS; 4 = one can get AIDS by sharing drug needles). The total number of correct answers to these twelve sex knowledge questions reflected participants’ sex-related knowledge, with higher scores indicating increased knowledge. The internal consistency reliability for this scale was acceptable (α = .88).

Participants’ sexual attitudes were assessed through 5 items: (a) premarital sex; (b) premarital pregnancy; (c) any kinds of mutually agreed upon sex; (d) compensated dating; and (e) multiple sex partners (1 = *accept*, 2 = *neutral*, 3 = *not accept*).

Six items assessed premarital sex and high-risk sexual activities: (a) whether they ever had sexual intercourse (*yes* or *no*); (b) total number of sexual partners in the past six months (coded as *zero*, *one*, or *more than one*); (c) the relationship category of their sexual partner in the past six months (with live-in partner, fiancé(e), or serious boy/girlfriend classified as *noncasual partner*; ordinary friend, cyber friend, stranger, or prostitute as *casual partner*); (d) whether contraceptives were used with their sexual partner in the past six months (*yes* or *no*); (e) whether they had been forced into sex or forced someone into having sex (*yes* or *no*); and (f) whether they had been pregnant (*yes* or *no*).

Contraceptive use included how consistently sexually active youth practiced contraception and what methods they used. Consistency of use was indicated by a multinomial variable (*use every time*, *use nearly every time*, *use more than half*, *use nearly half*, *use less than half*, *seldom use*). Methods of contraception included male and female condom, contraceptive pills, injection, rhythm, and coitus interruption. Perceived adequacy of contraceptive knowledge and reasons for not using contraception was also assessed.

### Data analysis

The analysis was conducted in two parts using SPSS 16.0 software (Chicago, IL: SPSS Inc.). First, descriptive statistics were conducted to provide profiles of the participants, sex-related knowledge, attitudes toward premarital sex, pregnancy, compensated sex, multiple sex partners, contraceptive use, and high-risk sexual behaviors. Next, gender differences of these variables were tested using chi-square tests for categorical variables and t-tests for continuous variables.

Second, four multivariate logistic regressions were performed on the sample to identify factors associated with high-risk sexual behaviors, including premarital sex, casual sex relationships, multiple sexual partners, and pregnancy. These high-risk sexual behaviors served as the dependent variable(s) and the model included demographic variables (e.g., age, gender, education, immigration status, parents’ marital status, and current study status), family life satisfaction, sex-related knowledge, and corresponding attitudes toward sex. Both odds ratio (OR) and its 95% confidence level interval (CI) were provided, and an alpha level of .05 was set for bivariate comparisons and multivariate analyses.

## Ethical considerations

This study has been approved by the Ethics Committee of the Faculty of Social Science, The University of Hong Kong.

## Results

### Socio-demographic characteristics of the respondents

As shown in Table 1, 594 (52.7%) of the youth were male, with an average age of 22.30 years (*SD* = 2.89). With regard to age range, 44% were between 18 and 21 years old, 27.5% were between 22 and 24 years old, and 28.3% were between 25 and 27 years old. Approximately 17% of the male young adults were born outside of Hong Kong. Only about 27.2% had received university or higher education, and 63.7% of the male were not in the school system. About 11.7% of the youth reported coming from a divorced family and 7.7% of them reported that either one or both of their parents were deceased. About 31% reported a less than satisfying family life.

**Table 1:** Characteristics of respondents

**Variable**	**Male**	**Female**	**Total**	**Chi-square or T tests**
N	594	532	1126	
Mean age (M ± SD)	22.30 ± 2.90	22.09 ± 2.87	22.20 ± 2.89	1.20
Age group				0.54
18-21	44.2	45.9	45.0	
22-24	27.5	27.6	27.5	
25-27	28.3	26.5	27.4	
Immigration status				1.27
Hong Kong	82.8	80.2	81.5	
Non-Hong Kong	17.2	19.8	18.5	
Education				13.40**
Junior high or lower	37.0	27.4	32.3	
Senior high	36.1	44.9	40.1	
College or higher	26.9	27.7	27.2	
Current school status				0.74
In school	36.3	38.8	37.4	
Out of school	63.7	60.9	62.6	
Marital status of parents				0.37
Married	80.4	81.0	80.6	
Divorced	11.5	11.9	11.7	
Deceased	8.1	7.2	7.7	
Family life satisfaction				12.76**
Very satisfied	47.8	51.3	49.5	
Satisfied	17.3	22.6	19.8	
Less than satisfied	34.9	26.1	30.7	

**p < .01.

Compared to male youths, female youths showed similar demographic characteristics in terms of age, immigration status, current school status, and parents’ marital status. However, they had higher educational attainment level (χ^*2*^ = 13.40, *p* < .01), and also reported more satisfaction with their family life than their male counterparts (χ^*2*^ = 12.76, *p* < .01).

### Sex-related knowledge and attitudes toward high-risk sexual behavior

As shown in Table 2, the youth demonstrated an adequate level of sex-related knowledge in general. On average, young adults correctly answered 10 of 12 knowledge items, with higher levels of knowledge concerning HIV/STD transmission (*M* = 3.55, *SD* = 0.84) and lower levels of knowledge about contraception (*M* = 3.07, *SD* = 1.10). Male youth reported higher sex-related knowledge, but the gender difference was not statistically significant.

**Table 2:** Sex-related knowledge and attitudes toward high-risk sexual behaviors among unmarried youth in Hong Kong

**Knowledge and attitudes**	**Male (n = 594)**	**Female (n = 532)**	**Total (n = 1126)**	**Chi-square or T tests**
Sex-related knowledge (M ± SD)	10.15 ± 2.17	9.98 ± 2.51	10.06 ± 2.36	1.17
Contraception (M ± SD)	3.09 ± 1.06	3.06 ±1.13	3.07 ±1.10	0.59
Sexually transmitted disease (M ± SD)	3.46 ± 0.83	3.39 ± 0.98	3.43 ± 0.91	1.36
HIV/STI transmission (M ± SD)	3.56 ± 0.81	3.54 ± 0.88	3.55 ± 0.84	0.39
Attitudes toward premarital sex				29.95***
Accept	69.5	57.3	63.8	
Neutral	20.4	21.2	20.8	
Not accept	10.1	21.4	15.5	
Attitudes toward any kinds of sexual activities that are mutually agreed upon				32.20***
Accept	61.1	45.9	53.9	
Neutral	20.5	22.7	21.6	
Not accept	18.4	31.4	24.5	
Attitudes toward premarital pregnancy				13.79**
Accept	52.5	43.6	48.3	
Neutral	19.4	18.0	18.7	
Not accept	28.1	38.3	32.9	
Attitudes toward compensated dating with sex				95.16***
Accept	24.6	7.3	16.4	
Neutral	20.4	11.1	16.0	
Not accept	55.1	81.6	67.6	
Attitudes toward multiple sex partners				89.63***
Accept	33.2	13.3	23.8	
Neutral	20.2	13.0	16.8	
Not accept	46.6	73.7	59.4	

**p < .01 ***p < .001.

The majority of the young adults (63.8%) reported acceptance of premarital sex by other people, 20.8% of them held neutral attitudes on the topic, and only 15.5% reported that they could not accept others engaging in premarital sex. In addition, over half of the young adults (53.9%) reported acceptance of any kind of sexual activity that was mutually agreed upon by other people, 21.6% of them held neutral attitudes, and 24.6% reported they could not accept mutually agreed upon sexual activities. Less than half of the young adults (48.3%) reported that they could accept premarital pregnancy, 18.7% held neutral attitudes, and 32.9% could not accept premarital pregnancy.

Although most youth held liberal attitudes toward premarital sex, any kind of mutually agreed upon sex activities, and premarital pregnancy, they were less tolerant of compensated dating with sex and multiple sex partners. Specifically, only 16.4% accepted compensated dating with sex and 23.8% accepted multiple sex partners. Compared to females, male youth reported more liberal attitudes towards premarital sex (69.5% vs. 57.3%), any kind of mutually agreed upon sexual activities (61.1% vs. 45.9%), premarital pregnancy (52.5% vs. 43.6%), compensated dating (24.6% vs. 7.3%), and multiple sex partners (33.2% vs. 13.3%).

### Premarital sex, high-risk sexual behavior and contraceptive use

Table 3 shows that 41.5% of the young adults reported that they had engaged in sexual intercourse. Males reported a higher prevalence of sexual intercourse than females (47.1% vs. 35.2%, χ^*2*^ = 16.81, *p* < .001). The mean age of first sexual intercourse was about 18 years old (*SD* = 2.00). With regard to multiple sexual partners, 6.4% reported that they had sex with multiple partners in the past six months, and males reported a higher prevalence of multiple sex partners than females (9.1% vs. 2.3%, χ^*2*^ = 17.90, *p* < .001). A small percentage of the youth engaged in a casual relationship in the past six months (2.4%), with males more likely to report engaging in this behavior (3.9% vs. 0.8%, χ^*2*^ = 8.06, *p* < .01). Only 2.7% of the youth reported that they did not use any contraceptive methods when they engaged in sexual intercourse and there was no gender difference on this issue. Also, 0.3% of the youth reported that they once forced their partner into sex, with no gender differences detected. 1.3% of youth reported that they were forced into sex by their partner, but females reported a higher prevalence of being forced into sex than males (2.3% vs. 0.8%, χ^*2*^ = 6.52, *p* < .05). Finally, 4.2% reported that they were involved in a pregnancy and the mean age of pregnancy was 19.26 years (*SD* = 2.10).

**Table 3:** Self-reported high-risk sexual behaviors among unmarried youth in Hong Kong

	**Male**	**Female**	**Total**	**Chi-square or T tests**
Lifetime sexual intercourse (n = 445)	47.1	35.2	41.5	16.81***
Mean age of first sexual intercourse (M ± SD)	17.72 ± 1.98	17.76 ± 1.94	18.00 ± 2.00	-0.22
Multiple sex partners in the past six months (n = 72)	9.1	2.3	6.4	17.90***
Casual sex relationship in the past six months (n = 27)	3.9	0.8	2.4	8.06**
No contraceptive methods used (n = 32)	3.9	1.3	2.7	3.34
Force partner into sex (n = 3)	0.5	0.2	0.3	2.69
Being forced into sex (n = 15)	0.8	2.3	1.3	6.52*
Being involved in a pregnancy (n = 47)	4.8	3.4	4.2	1.37
Mean age of pregnancy (M ± SD)	19.04 ± 2.02	19.58 ± 2.22	19.26 ± 2.10	- 0.86

*p < .05 **p < .01 ***p < .001.

As presented in Table 4, 90.6% of sexually active youth perceived that they had adequate contraceptive knowledge, 3.6% did not know, and only 5.7% reported inadequate knowledge, with no gender differences.

**Table 4:** Contraceptive methods used among sexually active unmarried youth

	**Male**	**Female**	**Total**	**Chi-square tests**
Adequacy of contraceptive knowledge (n)	314	153	467	7.47
Adequate	89.6	92.0	90.6	
Neutral	4.6	2.2	3.6	
Inadequate	5.7	5.8	5.7	
Frequency of contraceptive use in the past six months (n)	200	120	320	1.98
Uses each time	49.0	49.2	49.1	
Uses nearly each time	30.0	30.8	30.3	
Uses more than half	9.5	10.0	9.7	
Uses nearly half	3.0	4.2	3.4	
Uses less than half	3.0	0.8	2.2	
Seldom uses	5.5	5.0	5.3	
Contraceptive methods being used in the past six months (n)	183	131	314	14.35**
Male condom	96.2	84.7	91.4	
Female condom	1.1	3.8	2.2	
Contraceptive pills	2.7	6.1	4.1	
Injection	-	1.5	0.6	
Rhythm	-	0.8	0.3	
Coitus interruption	-	2.3	1.0	
Reasons for no contraception (n)	129	93	222	15.24
Have not thought of any sexual relationship before	54.3	45.2	50.5	
Don’t want to have any contraception	6.2	5.4	5.9	
It is shameful to buy contraceptive products	3.1	2.2	2.7	
I am afraid that my parents notice the contraceptive product	0.8	1.1	0.9	
I am afraid that contraception is harmful to health	0.0	3.2	1.4	
I thought my partner is responsible for the contraception	1.6	10.8	5.4	
I have not thought of contraception to be made	9.3	6.5	8.1	
Sexual intercourse only within the rhythm	12.4	9.7	11.3	
Don’t worry about getting pregnant	12.4	16.1	14.0	

**p < .01.

Regarding the practice of contraceptive use, less than half of sexually active youth in the past six months reported they used a contraceptive method every time, 30.3% used them nearly every time, about 15% used them around half of the time, and 5.3% seldom used any method. Male and females did not show any differences in the frequency of contraceptive use in the past six months.

Using a male condom was the most popular method of contraception reported (91.4%). Other methods were used less often, such as contraceptive pills (4.1%) and female condoms (2.2%). The primary reason reported by youth as to why they did not use contraception was that they had not thought the relationship would become sexual (50.5%). Other reasons included, “not worrying about pregnancy” (14%) and only engaging in sex “within the rhythm” (11.3%), with no gender differences present.

### Factors associated with high-risk sexual behavior

As shown in Table 5, with each year of age the likelihood of premarital sex increased 17% (OR = 1.17, 95% CI = 1.09 - 1.23). Females were 26% less likely than males to engage in premarital sex (OR = 0.74, 95% CI = 1.09 - 1.23). Immigrants were nearly 40% less likely to engage in premarital sex than local youth (OR = 0.60, 95% CI = 0.41 - 0.90). Young adults with higher education levels were nearly 50% less likely to engage in premarital sex than those with junior high or lower education levels (OR = 0.43, 95% CI = 0.29 – 0.62 [for senior high education]; OR = 0.50, 95% CI = 0.33 – 0.75 [for college or higher education] ). Youths from a divorced family were 60% more likely to engage in premarital sex (OR = 1.60, 95% CI = 1.00 - 2.55) than those from an intact family. Out of school youth were nearly 90% more likely to engage in premarital sex than those in school (OR = 1.89, 95% CI = 1.26 - 2.83). Favorable attitudes toward premarital sex were positively associated with premarital sex (OR = 6.70, 95% CI = 4.10 - 10.96).

**Table 5:** Associations between various characteristics and premarital sex and high-risk sexual behaviors

	**Premarital sex**		**Casual relationships**		**Multiple sex partners**		**Pregnancy**	
	**( n = 1117)**		**(n = 1120)**		**(n = 1125)**		**(n =1124 )**	
	**OR**	**95% CI**	**OR**	**95% CI**	**OR**	**95% CI**	**OR**	**95% CI**
Age	1.17***	1.09-1.25	1.20	0.93-1.49	0.89	0.78-1.02	1.15	0.98-1.35
Gender	0.74*	0.55-0.99	0.42	0.12-1.41	0.50	0.23-1.05	0.84	0.39-1.82
Immigration status								
No	1.00		1.00		1.00		1.00	
Yes	0.61*	0.41-0.90	3.24*	1.12-9.31	2.58*	1.13-5.92	1.92	0.76-4.86
Education								
Junior high or lower	1.00		1.00		1.00		1.00	
Senior high	0.43***	0.29-0.62	0.22	0.05-1.02	0.38*	0.16-0.88	0.17**	0.05-0.59
College or higher	0.50***	0.33-0.75	0.19*	0.04-0.84	0.50	0.21-1.19	0.23**	0.08-0.65
Marital status of parents								
Married	1.00		1.00		1.00		1.00	
Divorced	1.60*	1.00-2.55	0.80	0.19-3.32	0.50	0.16-1.36	1.06	0.34-3.29
Deceased	0.86	0.49-1.51	1.02	0.22-4.65	1.37	0.50-3.71	0.54	0.12-2.48
Studying status								
In school	1.00		1.00		1.00		1.00	
Out of school	1.89**	1.26-2.83	0.31	0.06-1.62	1.07	0.39-2.92	2.38	-
Family life satisfaction								
Very satisfied	1.00		1.00		1.00		1.00	
Satisfied	1.14	0.77-1.68	0.43	0.11-1.67	1.13	0.47-2.71	1.61	0.52-5.01
Less than satisfied	1.21	0.78-1.90	0.84	0.21-3.29	1.80	0.70-4.66	1.27	0.36-4.42
Sex-related knowledge	1.01	0.95-1.08	0.99	0.82-1.19	1.02	0.89-1.15	1.27	0.98-1.64
Attitudes toward high-risk sexual behaviors								
Not accept	1.00		1.00		1.00		1.00	
Neutral	0.98	0.54-1.79	1.88	0.48-7.26	3.43*	1.24-9.45	0.82	0.19-3.42
Accept	6.70***	4.10-10.96	3.36*	1.15-9.80	9.75***	4.15-22.90	1.98	0.76-5.15

Note: Attitudes towards high-risk sexual behavior in the regression model corresponded with a particular sexual behavior. For example, attitudes toward premarital sex were used to predict premarital sex, and attitudes toward compensated dating with sex were used to predict casual sex relationships.

*p < .05 **p < .01 ***p < .001.

Immigrant young adults were over three times likely to engage in a casual relationship than local youth (OR = 3.23, 95% CI = 1.12 - 9.31). Moreover, those with a university or higher education level were about 80% less likely to engage in a casual relationship than those with a junior high or lower education level (OR = 0.19, 95% CI = 0.04 - 0.84). Favorable attitudes toward compensated dating with sex were positively associated with casual sex relationships (OR = 3.36, 95% CI = 1.15 - 9.80).

Immigrants were over twice likely to engage in sex with multiple partners than local youth (OR = 2.58, 95% CI = 1.13 - 5.92). Furthermore, youth with senior high education levels were about 60% less likely to engage in multiple sexual relationships than those with junior high or lower education levels (OR = 0.38, 95% CI = 0.16 - 0.88). Neutral and favorable attitudes toward multiple sex partners were positively associated with multiple sex partners (OR = 3.43, 95% CI = 1.15 - 9.80 [for neutral attitudes]; OR = 9.75, 95% CI = 4.15 - 22.90 [for favorable attitudes]). Youth with higher education levels were about 80% less likely to experience premarital pregnancy than those with junior or lower education levels (OR = 0.17, 95% CI = 0.05 - 0.59 [for senior high education]; OR = 0.23, 95% CI = 0.08 - 0.65 [for college or higher education]).

## Discussion

Using a representative sample of 1,126 youth in Hong Kong, this study investigated unmarried youth’s sex knowledge, attitudes, and high-risk sexual behaviors. In general, Hong Kong unmarried youth showed adequate knowledge in STD and HIV/STD transmission, but contraception knowledge was relatively inadequate. Although the majority of youth perceived that they had adequate contraceptive knowledge, less than half had consistently used any contraceptive methods during sexual intercourse. Half of the sexually active youth who did not use contraceptive methods indicated that sexual intercourse happened unexpectedly, so they had no time to plan the contraception issues. It is also possible that some men may not care about their female partner’s pregnancy. Although the Hong Kong Government has published guidelines on sex education, sexual matters continue to be taboo topics in formal settings, thus knowledge on contraception has not been provided through official channels [23]. The fact that over twelve percent of our unmarried youth who did not use contraception reported they engaged in sexual intercourse only within the rhythm and over twelve percent of them were not afraid of getting pregnant demonstrated that contraception should be appropriately addressed in the development of sex education and HIV prevention programs. In sum, it is important to focus contraception efforts on unmarried youth.

Over half of Hong Kong unmarried youth showed liberal attitudes toward premarital sex and any kinds of sexual behavior that are mutually agreed upon, which is lower than their counterparts in the United States [24], but higher than those reported by Hong Kong university students [13]. Nearly half of our youth had favorable attitudes towards premarital pregnancy, which is much higher than that of out of school youth between 16 and 24 years old in Shanghai, the most westernized city in Mainland China [21]. However, over 83% of these unmarried Hong Kong youth had unfavorable attitudes towards compensated dating and over 76% had unfavorable attitudes towards multiple sex partners, consistent with the low prevalence of multiple sex partners among female college students in China [25]. In contrast, one study on young people in the United States, 31.1% of sexually experienced females and 45.0% of sexually experienced males reported that they had six or more sex partners by age 21 [26]. It seems that despite the long history of Western influence, Hong Kong youth still maintain some conservative attitudes toward sex. In addition, consistent with earlier studies [9,16], males showed more liberal and acceptance attitudes toward premarital sex and other high-risk sex behaviors than females.

The prevalence of premarital sex among Hong Kong unmarried youth was 41.5%. This figure has increased significantly compared to the same age group fifteen years ago [3]. However, the prevalence of more risky sex behaviors (e.g., multiple sex partners, casual relationships, no contraceptive methods used, sex coercion, and pregnancy) was much lower than premarital sex. These findings indicate that although liberal attitudes toward premarital sex seem quite natural since Hong Kong has been undergoing rapid westernization, when compared to other Asian societies the Hong Kong Chinese culture values conformity and has a low tolerance for rule violation among young adults [14]. Similar to a previous study, gender differences in premarital sex, multiple partners, and casual relationships were present, with males reporting more sexual activity and risky sex behaviors [3]. However, females reported more sexual coercion than males. This may reflect that females are more likely to be the victim in a sexual relationship.

Finally, consistent with previous studies [17,21], this study showed that older age, being male, coming from a divorced family, and being out of school were all associated with having experienced sexual intercourse. Being better educated is a protective factor for high-risk sex behavior as well as premarital sex, suggesting that the school environment might provide a context where early sexual activities are discouraged [27]. Similar to previous studies, liberal attitudes toward premarital sex and high-risk sex behaviors are prominent factors associated with corresponding sex behavior [21,28]. Sex knowledge seems to be less important to sex behavior, and one previous study found similar results [21]. There are two unexpected findings that are discussed in turn. First, immigrant youth were less likely to engage in premarital sex, but were more likely to have casual relationships and have multiple sex partners. One plausible explanation is that immigrant youth may be conservative for premarital sex, and liberal attitudes for casual sex and multiple sex partners, which should be explored in future studies. Second, family life satisfaction showed no association with any high-risk sex behaviors, which is inconsistent with the previous findings [29]. However, one study on college students in India showed that family environment had no significant influence on their sexual experience [30]. It may suggest that eastern family is losing its traditional control over young adults’ sexual behavior. Meanwhile, it should be noted that only one global measurement item might fail to capture the full dimensionality of family life, so multiple items could be used in future studies.

There are several limitations that should be highlighted. First, it was a cross-sectional survey, thus time sequence and cause-effect relationships among these variables could not be established. Second, although anonymity and confidentiality were emphasized during data collection, premarital sex and high-risk sex behavior are socially unacceptable in Hong Kong, so the prospect of underreporting should be acknowledged.

## Conclusions

Based on a representative sample of unmarried youth, this study reports young adults’ sex knowledge, attitudes, and high-risk sexual behaviors in contemporary Hong Kong. Our findings have implications for future sex education, policymaking, and HIV prevention programs. First, unmarried youth should be targeted with an emphasis placed on contraception and HIV knowledge, since youth are not adequately versed in these domains of sex knowledge. Second, they should be informed about the negative consequences associated with liberal attitudes toward premarital sex and high-risk sex behavior, thus those youth with neutral attitudes will be more likely to change their opinions. Third, more attentions should be paid to high-risk groups, such as those youth who are older, male, immigrants, come from a divorced family, and are currently out of school.

### Consent

Written informed consent was obtained from the participant for the publication of this report and any accompanying images.

## Abbreviations

STI: Sexually transmitted infection; STD: Sexually transmitted disease; HIV: Human immunodeficiency virus.

## Competing interests

The authors declare that they have no competing interests.

## Authors’ contributions

PSFY conceived, designed and supervised all aspects of this research and statistical analysis. HZ performed the data analysis and drafted and revised the manuscript. THL participated in the interpretation of the results and made contributions to the revisions of the manuscript. KFL involved in statistical analysis and results interpretation. JC and SF implemented this research and made contributions to the edit of the manuscript. All authors read and approved the final manuscript.

## Pre-publication history

The pre-publication history for this paper can be accessed here:

http://www.biomedcentral.com/1471-2458/13/691/prepub
